# Mycobacterium-Induced Th1, Helminths-Induced Th2 Cells and the Potential Vaccine Candidates for Allergic Asthma: Imitation of Natural Infection

**DOI:** 10.3389/fimmu.2021.696734

**Published:** 2021-08-03

**Authors:** Mohamed Hamed Abdelaziz, Xiaoyun Ji, Jie Wan, Fatma A. Abouelnazar, Sayed F. Abdelwahab, Huaxi Xu

**Affiliations:** ^1^International Genomics Research Center (IGRC), Institute of Immunology, Jiangsu University, Zhenjiang, China; ^2^Department of Microbiology and Immunology, Faculty of Medicine, Al-Azhar University, Assiut, Egypt; ^3^Department of Neuroimmunology, Brigham and Women’s Hospital, Harvard Medical School, Boston, MA, United States; ^4^Department of Clinical Laboratory Diagnostics, School of Medicine, Jiangsu University, Zhenjiang, China; ^5^Division of Pharmaceutical Microbiology, Department of Pharmaceutics and Industrial Pharmacy, College of Pharmacy, Taif University, Taif, Saudi Arabia

**Keywords:** allergy, asthma, BCG, helminths, immunotherapy, mycobacteria, Th1/Th2, vaccines

## Abstract

Bronchial asthma is one of the most chronic pulmonary diseases and major public health problems. In general, asthma prevails in developed countries than developing countries, and its prevalence is increasing in the latter. For instance, the hygiene hypothesis demonstrated that this phenomenon resulted from higher household hygienic standards that decreased the chances of infections, which would subsequently increase the occurrence of allergy. In this review, we attempted to integrate our knowledge with the hygiene hypothesis into beneficial preventive approaches for allergic asthma. Therefore, we highlighted the studies that investigated the correlation between allergic asthma and the two different types of infections that induce the two major antagonizing arms of T cells. This elucidation reflects the association between various types of natural infections and the immune system, which is predicted to support the main objective of the current research on investigating of the benefits of natural infections, regardless their immune pathways for the prevention of allergic asthma. We demonstrated that natural infection with *Mycobacterium tuberculosis* (Mtb) prevents the development of allergic asthma, thus Bacille Calmette-Guérin (BCG) vaccine is suggested at early age to mediate the same prevention particularly with increasing its efficiency through genetic engineering-based modifications. Likewise, natural helminth infections might inhabit the allergic asthma development. Therefore, helminth-derived proteins at early age are good candidates for designing vaccines for allergic asthma and it requires further investigation. Finally, we recommend imitation of natural infections as a general strategy for preventing allergic asthma that increased dramatically over the past decades.

## Background

Bronchial asthma is regarded as the most chronic pulmonary disease and a major public health problem, affecting more than 350 million people worldwide with a high mortality rate in severe cases. Also, it is likely to afflict an additional 100 million by 2025. Among the various forms of asthma (minor forms due to air pollution, exercise, aspirin and cold), allergic or atopic asthma is the most prevalent (1, 2). T helper 2 (Th2) cells are the major effector cells in the pathogenesis of allergic asthma. Upon allergic exposure and *via* their signature cytokines, they stimulate Eosinophils and IgE-producing B cells with subsequent mast cells degranulation, resulting in the characteristic airway narrowing and airway hyperreactivity (AHR) (2). Generally, asthma is more prevalent in developed countries (range between 8.5% to 32%) than in developing countries (range between 4.1% to 4.2%) and its prevalence is increasing in the developing countries as they become more westernized (1, 3). The first study investigated this phenomenon was in 1976, when the authors found that allergic diseases increase more in urban areas than in rural areas, which resulted from less exposure to viruses, bacteria and helminths in urban areas (4). In 1989, a study reported similar findings, and concluded that higher household hygienic standards lead to decreased chances of infections, which may subsequently increase the occurrence of allergy, and it has been referred as the *hygiene hypothesis* (5). This hypothesis suggest that the removal of the regulatory effects of infectious microorganisms from populations tended to lead to an imbalance in the immune system (6), which acts through two patterns of acquired immune response: Th1 and Th2 immune responses (7). In addition, over the past 20–30 years, it has become increasingly clear that, in Western countries, a strong correlation exists between improved sanitation and hygiene and a dramatic increase in atopic disorders (8). Moreover, this hypothesis was supported by the global rise in allergy associated with a decreased infection burden (1, 6, 9–11). This hypothesis has been extended to include different autoimmune diseases, such as type I diabetes mellitus, inflammatory bowel disease, and multiple sclerosis (8, 11, 12). Moreover, there is another hypothesis derived from the *hygiene hypothesis* and called old friends that implies the relationship between various types of infections and chronic inflammatory diseases (13). Furthermore, the biodiversity hypothesis, another hypothesis derived from the *hygiene hypothesis*, demonstrates that reduced natural environmental biodiversity adversely affects human commensal microbiota, which is consequently associated with higher prevalence of atopy (14). In general, these illustrations indicate the significance of the *hygiene hypothesis* to gain further knowledge concerning allergic asthma.

The main attempt of the current study was to integrate existing knowledge with the *hygiene hypothesis* to attain beneficial preventive approaches for allergic asthma. Therefore, we highlighted the studies that illustrated the correlation between allergic asthma and two different types of infections (mycobacteria and helminths) that induce the two major antagonizing arms of T cells (Th1 and Th2). This elucidation reflects the relationship between various types of natural infections and the immune system, which consequently supports our main attempt to investigate the potential advantages of various natural infections and the practical applications of these benefits for developing efficient approaches to control allergic asthma.

## Mycobacterium tuberculosis and Allergic Asthma

Tuberculosis (TB) is a highly infectious granulomatous lung disease induced by *Mtb*, affects 10 million new cases annually with 1.5 million associated deaths predominantly in developing countries, representing the most lethal pathogenic organism worldwide (15). *Mtb* is a potent Th1 response inducer. After *Mtb* reaches the respiratory tract, it is deposited within the alveoli, to which the immune system responds by releasing pro-inflammatory cytokines that recruit monocytes and macrophages. *Mtb* begins to multiply within alveolar macrophages secreting interleukin (IL)-12, the latter activates IFN-γ-secreted Th1, and thus activates macrophages and enhances its intracellular killing of phagocytosed *Mtb*. This immune response can control the infection in 90% of cases. However, *Mtb* are not completely eradicated and their intracellular persistence inside macrophages induces Th1 hypersensitivity, that stimulate the formation of chronic granuloma, which is a structure consisting of a central zone of multinucleated giant cells containing the organisms, in addition to the peripheral zone of fibroblasts, lymphocytes and monocytes to limit bacterial spread (15). Th1-directed response is mainly induced by certain antigenic structures of cell wall of *Mtb*, such as the mycolyl arabinogalactan peptidoglycan complex and their associated lipoarabinomannan. These structures give *Mtb* their acidic stability and prolong their survival inside macrophages (16). Notably, T-regulatory cells (Tregs) are involved in the immune response to *Mtb via* potentially suppressing the pathogenic hyperactivation of Th1 cells (17, 18).

In an asthmatic mice model, *Mtb* infection alleviates allergic inflammation and reduces Th2 cytokines. These effects were suggested to be mediated through the conversion of the allergen-specific Th2 into Th1 cells, which was supported by increased IFN-γ secretion of allergen-specific T cells, and not by *Mtb*-stimulated expanded T cells. In addition, this switch was induced by IL-12, which is the classical cytokine associated with *Mtb* infection (15, 19). These findings indicate an inverse correlation between murine experimental allergic asthma and *Mtb* infection. Concurrently, for human allergic asthma, an international ecological study was conducted by the International Study of Asthma and Allergies in Childhood (ISAAC), using data from 23 countries in Europe, USA, Canada, Australia and New Zealand, and found that childhood *Mtb* infection may protect against the subsequent development of allergic asthma (20). Another ecological study included world health organization and ISAAC program data for standardized asthma symptoms and TB prevalence collected from Asian, central and south American, and African countries. This study concluded a preventive effect of *Mtb* infection against the development of asthma through the induction of strong Th1 immune response (21). Moreover, the protective role of *Mtb* was concluded by other studies (22–24). Interestingly, as reported in mice, this effect might be induced by the switching of allergen specific Th2 into Th1 cells in human (23). Furthermore, there was an inverse association between tuberculin test positivity and the incidence of allergic asthma (25, 26). In general, early childhood *Mtb* infection can prevent the subsequent development of allergic asthma.

### BCG and Allergic Asthma

The protective role of *Mtb* infection against allergic asthma indicates the elucidation of the potential role of the TB vaccine in preventing asthma. The only licensed vaccine for TB is the BCG that is named after Albert Calmette and Camille Guérin who in 1908 attenuated the living virulent *mycobacterium bovis* (*M. bovis*) through continuous passages (231 passages) on culture media that lost the virulence and maintained the antigenicity of the organism. This vaccine was made throughout 13 years at the Institute Pasteur in Lille, France, and was used for the first time in humans in 1921 for TB prevention, with more than 100 million children vaccinated annually worldwide (27, 28). However, the protective efficacy of BCG is variable, ranging from 0% to 80% in different countries. Nonetheless, the exact reason for this variation remains unclear (27, 29, 30).

Interestingly, BCG is not a single strain, as the original strain in Pasteur institute generated several offspring strains *via* continuous passages in the countries that received the vaccine that reached 60 countries by 1927. For example, the Danish strain originated in 1931 in the name of 423rd transfer, whereas the Glaxo strain was derived from the 1077th transfer of the Danish strain. Many other strains were used, for instance; Pasteur, Tokyo, Prague, Russian, Moreau, …etc. (30, 31). The mechanism of the attenuation process of *M. bovis* resulting from the serial passages is not well interpreted. Nevertheless, it may involve deletion of a chromosomal region, called region of difference 1 (RD1), which is found only in virulent mycobacteria and absent in BCG, containing the encoding genes for ESAT-6 and CFP-10 and their secretion apparatus which are two fundamental virulence factors for *M. bovis*. Furthermore, other RD regions such as RD2, RD3 and RD14 to RD16 may be omitted. Moreover, single nucleotide polymorphisms are also involved in chromosomal changes (27, 28). Therefore, the genetic variability between the various BCG vaccine strains may explain the variable protection. Also, they induced different degrees of immune cell responses *in vitro*. However, there is no clear evidence that one strain produces more protection against pulmonary TB than the remaining strains (30). Another variable for the efficacy of BCG is the prior exposure to environmental mycobacteria, which are distributed differently among countries. These mycobacteria affect the efficacy of BCG through its pre-existing immune response that may block BCG replication, hence called the *blocking hypothesis*. In addition, it may induce a certain level of protection against TB, which masks any protective effect of subsequent BCG, thus called the *masking hypothesis* (28, 32).

BCG is considered very safe and the following reactions following intradermal (i.d.) administration are mild in the form of erythema and papule or ulceration that develops into scar, and generally do not require any treatment (33). BCG stimulates the immune response *via* binding its antigenic structures, such as lipoarabinomannan, phosphatidylinositol mannoside and trehalose 6,60-dimycolate, to toll-like receptor (TLR)2 and TLR4 on innate immune cells as macrophages that secrete IL-12 to induce Th1 cells polarization. The latter cells secrete IFN-γ that activates macrophages in a positive feedback loop (34, 35). Consequently, BCG is a potent inducer of Th1 cells which is, also, converted into memory cells to maintain life-long protection against *Mtb* infection. However, BCG, also, stimulates polarization of Tregs, as the response to *Mtb* infection, to prevent an exaggerated Th1 response (34). Due to the immunostimulatory effects of BCG, it is used as an immunotherapy for bladder cancer, multiple sclerosis and type 1 diabetes mellitus, and it also used as an adjuvant (35).

In experimental murine allergic asthma, we divided the potential effects of BCG into three categories (**Table 1**): 1) preventive, 2) preventive and/or therapeutic, and 3) therapeutic, according to three time sets of vaccine administration: 1) before allergen sensitization (36–43), 2) with allergen sensitization (44–46), and 3) with/after aerosol allergen challenge (47, 48), respectively. In several studies, administration of BCG after birth or at an early age prevents the subsequent development of murine allergic asthma regardless of strain, route and number of doses (36–46). In addition, the BCG protective response is supported by the ability of BCG adoptive transferred stimulated dendritic cells (DCs) with different subsets to suppress the established allergic inflammation in murine asthma (37, 49–51). Moreover, the BCG administration after the establishment of allergic asthma still has the potential to suppress allergic inflammation and AHR (47, 48), indicating that BCG is not only a preventive agent, but also a therapeutic candidate for allergic asthma. Correspondingly, for human allergic asthma (**Table 2**), several epidemiological studies with various study designs concluded that early BCG vaccination significantly decreases the subsequent development of asthma in different countries, such as England, France, Turkey, Germany and Spain in Europe, and Japan, Thailand and India in Asia (25, 52–57). Moreover, this conclusion was supported by the inverse correlation between BCG scar dimeter and atopic asthma in Brazil and Korea (65, 66). Interestingly, BCG scar diameter is a significant reflection of the immune response to BCG as a Th1 enhanced response with the subsequent increase of IFN-γ (66). Furthermore, BCG administration to asthmatic patients demonstrated therapeutic efficiency in the form of improved pulmonary functions and reduced medications. This occurs *via* the attenuation of Th2 response (67, 68), which proved the inhibitory effects of BCG even after developing atopic asthma.

**Table 1:** Impact of BCG vaccination on experimental murine allergic asthma.

Type of BCG strain	Strain condition	Animal type	Animal age	BCG administration route	Study times		The proposed effects	Proposed immune mechanisms compared to asthmatic mice	Study year	Reference
					Time of BCG vaccination	Asthma induction times				
Pasteur strain 1173P2	Live attenuated	BP2 mice	10 days (newborns)	i.n.	Day 0	- OVA sensitization: days 98 and 105 - Challenge: day 112	Preventive	↓ Eosinophilia, pulmonary inflammation and AHR - No change in IFN-γ	2001	(36)
					Day 28		No suppressive effects			
					Day 56					
Tokyo 172, Japan	Freeze-dried living	BALB/c mice	6 weeks	i.p.	Day 0	- OVA sensitization: days 5 and 12 - Challenge: days 19-21	Preventive	↓ Eosinophilia, pulmonary inflammation, OVA-specific IgE and AHR - The action depended on DCs-induced Tregs	2014	(37)
Pasteur F1173P2, Korea	Live attenuated	BALB/c mice	6 weeks	i.p.	Day 0	- OVA sensitization: days 7 and 21 - Challenge: days 28-30	Weak preventive effect	↑ IFN-γ/IL-5 ratio	2005	(38)
Tokyo 172, Korea							preventive	↓ Eosinophilia, IL-5 and AHR ↑ IFN-γ/IL-5 ratio ↓ IL-10		
Tice, Netherlands							Mild preventive	↓ Eosinophilia ↑ IFN-γ/IL-5 ratio		
Connaught, Canada								↓ Eosinophilia		
Moreau, Brazil	lyophilized	BALB/c mice	Newly weaned	i.d.	Day 0 or 30	- OVA sensitization: days 60, 67, 74, 81, 88 and 95 - Challenge: days 100-102	Preventive	↓ Eosinophilia, pulmonary inflammation, airway remodeling, IL-4, IL-5, IL-13 and AHR ↑ Tregs and IL-10	2013	(39)
				i.n.						
Moreau sub strain, Brazil	Live attenuated	BALB/c mice	10 days	i.n.	Day 0	- OVA sensitization: day 3 - Challenge: days 16, 17, 23 and 24	Preventive	↓ Eosinophilia, pulmonary inflammation, IL-4, IL-5, IL-25 And OVA-specific IgE ↑ IL-12 ↑ pulmonary DCs and its expression of TLR-2, TLR-4 and PD-L1 ↑ IL-10 and TGF-β	2017	(40)
Strains were obtained from; Behring, Marburg, Germany	Live attenuated	BALB/c mice	6 -8 weeks	i.v.	Day 0	- OVA sensitization: days 14, 28 and 35 - Challenge: days 40 and 41	Preventive	↓ Eosinophilia IL-4, IL-5 and OVA-specific IgE ↑ IFN-γ ↓ IL-10	1998	(41)
D2‐BP302, shanghai, China	Freeze‐dried living	C57BL/6 mice	Neonates	i.d.	Days 0, 7 and 14	- OVA sensitization: days 35 and 49 - Challenge: day 63	Preventive	↓ Eosinophilia, pulmonary inflammation, mucus overproduction IL-4, IL-5 and AHR ↑ IFN-γ	2008	(42)
						- OVA sensitization: days 35 and 49 - Challenge: day 315		↓ Eosinophilia, pulmonary inflammation and AHR - No changes in IL-4 nor IL-5		
D2-BP302, Shanghai, China	Freeze-dried living	C57BL/6 mice	Neonates	s.c.	Days 0, 7 and 14	- OVA sensitization: days 56 and 70 - Challenge: days 80-82	preventive	↓ Eosinophilia, pulmonary inflammation ↑ IFN-γ ↑ Th1 cells migration to the lung	2013	(43)
Tokyo 172	live attenuated	BALB/c mice	6 weeks	i.n.	Day 0	- OVA sensitization: days 0, 7, 14 and 21 - Challenge: days 28-30	Preventive and/or therapeutic effects with s.c. more than IN route	↓ Eosinophilia and AHR ↑ IFN-γ and IFN-γ/IL-5 ratio	2007	(44)
				s.c.						
Tokyo 172, Korea	Live attenuated	BALB/c mice	6 weeks	i.p.	Day 0	- OVA sensitization: days 0 and 14 - Challenge: days 21-23	Preventive and/or therapeutic	↓ Eosinophilia, AHR ↓ IL-17A ↑ IFN-γ/IL-5 ratio	2010	(45)
	Heat killed						Weak suppressive effects	Weak changes		
Strains obtained from Shanghai Research Laboratory of Biological Products	Inactivated	Sprague‐Dawley rats	4 weeks	i.d.	- Days 0 - continued 2 times each week, for a total of 9 weeks	- OVA sensitization: days 3 and 18 - Challenge: day 25, then 3 times each week, for a total of 6 weeks	Preventive and/or therapeutic	↓ pulmonary inflammation, airway re- modeling and AHR ↑ Tregs, TGF-β and CTLA-4 expression	2016	(46)
Tice; Organon, West Orange, NJ)	Lyophilized	BALB/c mice	4-5 weeks	i.n.	Day 33	- OVA sensitization: days 0, 14 and 28-30 - Challenge: days 32 and 40	Therapeutic	↓ Eosinophilia, IL-5 and AHR ↑ IFN-γ	2002	(47)
				i.p.			Weak suppressive effects	Weak changes		
Moreau sub-strain, Brazil	Live attenuated	BALB/c mice	6-8 weeks	i.n.	Days 35 and 42	- OVA sensitization: days 0 and 14 - Challenge: days 28-30, 34, 41, 63	Therapeutic	↓ Eosinophilia, pulmonary inflammation, IL-4, IL-13 and OVA-specific IgE ↑ IFN-γ ↑ Tregs, IL-10 (by CD8^+^) and TGF-β	2012	(48)

OVA, Ovalbumin; DCs, dendritic cells; i.d., intradermal; i.n., intranasal; i.p., Intraperitoneal; S.C., Subcutaneous.

**Table 2:** Impact of BCG vaccination on human allergic asthma.

Country	Study design	Type of strain	Age of BCG vaccination	No. of vaccinated subjects/total subjects	Age asthma diagnosis	Proposed effect against asthma	Notes	Study year	References
Japan	Retrospective	Tokyo 172 strain	At birth	867/867	12-13 years	Preventive	No significant difference between studied groups regarding family history	1997	(25)
Thailand	Prospective cohort	Strains were obtained from Thai Red Cross Society (Queen Soavabha Memorial Institute, Bangkok, Thailand)	Within the first 2 months	550/550	9-12 months -follow up at age of 2 years	Preventive		2004	(52)
Turkey		Freeze-dried, Pasteur Merieux (Lyon, France)		604/604					
France	Population based cohort	Not reported	First month	694/718	12-15 years	Preventive		2005	(53)
England	Retrospective cohort	Not reported	Before the age of 12 weeks (neonatal period)	1900/5086	6-11 years	Preventive	Family history of asthma significantly associated with an increased prevalence of asthma in children	2007	(54)
India	Cross-sectional	Not reporter	Early infancy	9492/10028	7-14 years	Preventive		2013	(55)
Germany	Cross-sectional	Copenhagen strain 1331	At neonatal period	20 383/38808	Mean age 6 years	Weak protective but significant		2002	(56)
Spain	Retrospective cohort	Copenhagen strain 1331, Pharmacia Upjohn	At birth	6762/9590	6-7 years	Weak protective but significant		2005	(57)
Sweden	retrospective cohort	Copenhagen strain 1331, Denmark	17-21 days (mean age)	216/574	5.5 years (mean age)	No correlation	No significant difference between vaccinated and control group regarding family history	1997	(58)
Sweden	cohort	Not reported	within first year of life	294/6497	4-9 years	No correlation		1998	(59)
Germany	Prospective cohort	Copenhagen strain 1331, Germany	Median age 30 days	92/774	Physical examination and history at 3, 6, 12, 18, 24, 36, 48, 60, 72 and 84 months	No correlation	- protection during first 2 years only - No significant difference between vaccinated and non-vaccinated group according to family history	2001	(60)
Germany and Netherlands	nested case-control	Not reported	At Infancy period	75/510	7-8 years	No correlation	BCG increase risk for HDM sensitization	2004	(61)
Germany	cross-sectional study	Not reported	Not reported	1219/1673	5-7 years	No correlation		2007	(62)
Netherland	randomized, prospective, single-blind study	Danish strain 1331	6 weeks	62/121	6 weeks, 4, 18 months	No correlation	No significant difference between studied groups regarding family history	2008	(63)
Canada	retrospective population-based birth cohort	Pasteur strain 568-571	32900 received at first year of life 2712 children received later	35612/ 76623	Followed until age of 20 years	No correlation	No significant difference between asthmatic and non-asthmatic subjects regarding family history	2017	(64)

**Figure 1:**
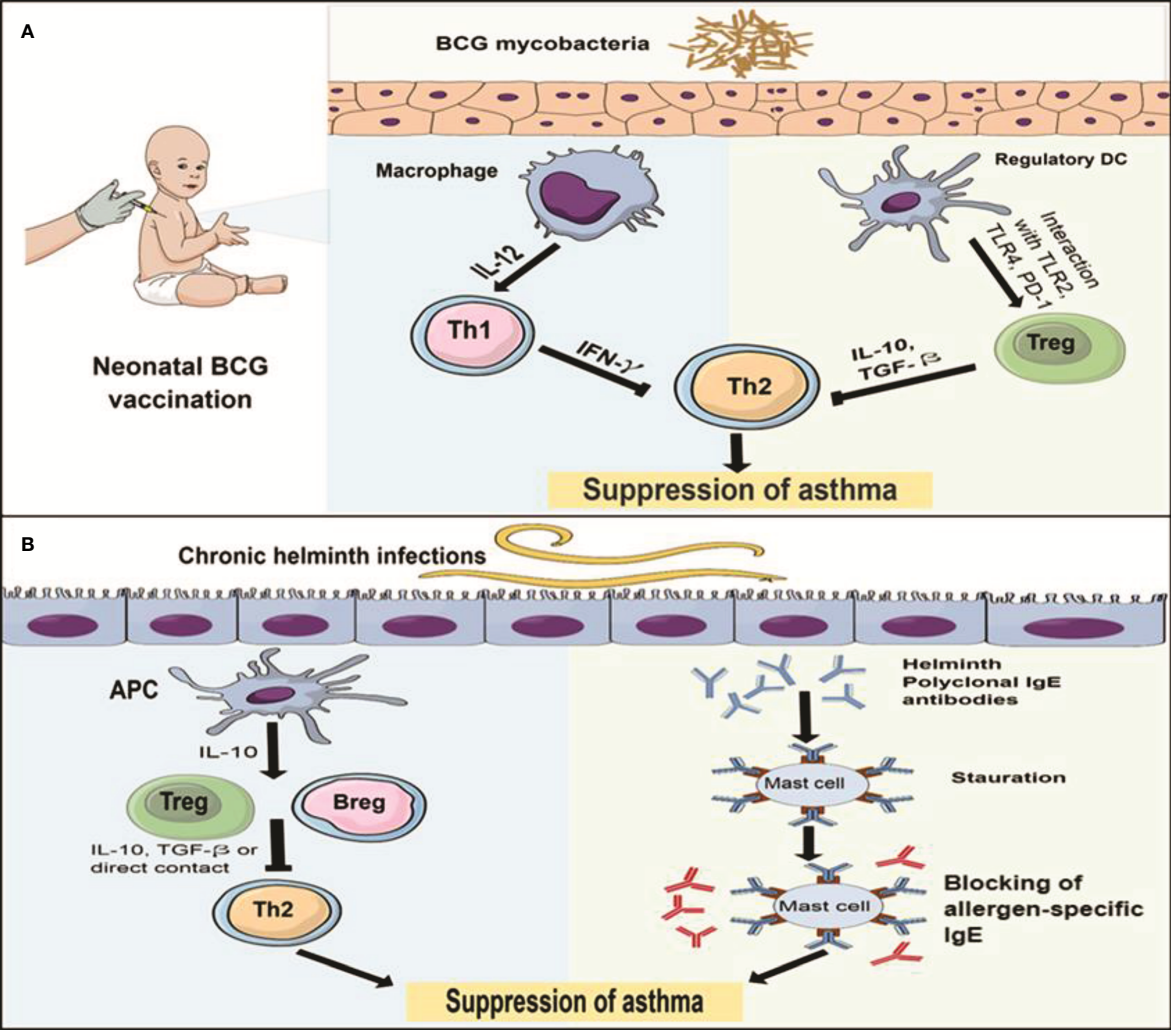
The immune mechanisms of BCG and helminths protect against allergic asthma **(A)** The protective effect of neonatal BCG is mediated through two pathways: the first pathway (left) is induction of Th1 cells polarization by IL-12 secreting macrophages, the resultant IFN-γ inhibits Th2 response with subsequent suppression of asthma. Another pathway (right) is the upregulation of Tregs polarization through interaction with TLR2, TLR4 and PD-1 expressed on DCs, the resultant IL-10 and TGF-β reduce TH2 response and suppress asthma. **(B)** The protective effect of chronic helminth infection is mediated through 2 pathways: the first pathway (left) is the induction of T and Bregs differentiation by IL-10 secreting antigen presenting cells (APCs). Then, Th2 response is inhabited by IL-10 and TGF-β or the direct contact between Tregs and Th2 cells, which leads to suppressing asthma. The second pathway (right) is the saturation hypothesis in which helminth infections induce polyclonal IgE (blue) that binds to and saturate high affinity FcϵR on mast cells, preventing binding of allergen-specific IgE (red) and subsequent blocking of mast cell degranulation.

The protective effect of BCG against allergic asthma can be mediated through two potential pathways (**Figure 1A**). First, promoted Th1 cells polarization with the subsequent increase in Th1/Th2 homeostasis along with their signature cytokines as IFN-γ/IL-5 ratio. This promotion could be induced by either polarization of naïve CD4+ to Th1 cells or switching of allergen specific Th2 into Th1 cells (38, 41–43). Another pathway is the upregulation of Tregs differentiation with the subsequent increase of IL-10, which is induced by interaction with stimulated TLR2, TLR4 and PD-1 that was expressed in DCs (37, 39, 40, 46). Interestingly, BCG can stimulate differentiation of naïve DCs into different subsets as CD8α^+^ and CD8α^-^ DCs that can induce both Tregs and Th1 cells through secretion of IL-10 and IL-12, respectively (37, 49–51). These two pathways provide protection in the form of suppression of Th2 cytokines secretion, eosinophilia, allergic inflammation, allergen specific-IgE and AHR (36–48). Moreover, BCG protected against asthma through reduced IL-17 production, the signature cytokine of Th17, that played a critical role in inducing neutrophilia and airway inflammation and correlated with AHR and disease severity (45, 69, 70). However, the complex action of Th17 in eosinophilic airway inflammation requires further elucidations.

On the contrary, several epidemiological studies found that early BCG vaccination did not decrease the risk of subsequent development of atopic asthma, these studies were performed in European countries such as Germany, Sweden, Netherland and a North American country; Canada (**Table 2**) (58–64). However, there weren’t adequate animal research that supported this claim unlike the BCG protective effects that were reinforced by several animal studies as mentioned previously. The discrepancy between the preventive role of BCG vaccination in the development of human asthma reported in different countries is attributed to some factors. First, BCG strains contrasted with different immunogenicity (28); however, as was shown in **Table 2**, the lack of information about the specific strains used in six of the 14 studies that supported (53–55) or did not support (59, 61, 62) the negative association between asthma and BCG, resulted in a severe difficulty to interpret the role of strains.

Nonetheless, four of seven studies (**Table 2**) supported the protective role of BCG against asthma and reported strains, such as Pasteur (52), Tokyo 172 (25) and Copenhagen 1331 strains (56, 57), while the latter induced poor protective effects. Interestingly, for the seven studies that did not support the protective role of BCG (**Table 2**), Copenhagen strain 1331 was utilized in three studies in Germany (60), the Netherlands (63) and Sweden (58). However, the other three studies, which did not mention the strain were also performed in the same three countries (59, 61, 62), with some potential to use the Copenhagen strain. The effect of this particular strain has not been investigated through animal research regarding its effect on murine allergic asthma unlike other strains shown in **Table 1**. Thus, among the different BCG strains, Copenhagen strain may not induce protection against allergic asthma, and this requires further investigations. In addition, the seventh non-supportive study used the Pasteur strain and was conducted in Canada (64). However, the study did not exclude the subjects who received the vaccine after the neonatal period. Consequently, this leads to the second point of the controversy, which is that late administration of BCG during the neonatal period may increase the chances of prior exposure to environmental mycobacteria, that might decrease the efficacy of BCG as previously mentioned. In same context, another study was implemented in Sweden that did not exclude receivers (59).

Atopic hereditary can potentially interfere with the effects of BCG, particularly in the seven studies that did not support the protective function. Four of these seven studies did not detect a significant difference between vaccinated and non-vaccinated or asthmatic and non-asthmatic subjects regarding family history of atopy (58, 60, 63, 64). Three other studies did not apply this comparison, which weakens the potential interference of the genetic background with the BCG effects in those studies. In contrast, studies demonstrating the protective effects of BCG on allergic asthma in developed countries such as England and France (53, 54), which consequently contradicts the assumption that the preventive effects of BCG against asthma are mediated by the natural infection with *Mtb* in developing countries, where the incidence of tuberculosis is high, and not by the BCG itself (58). However, the preventive role of *Mtb* infection against the development of asthma, which was previously mentioned, is not controversial as BCG. Interestingly, *Mtb* infection generates four times more IFN-γ than BCG (45). Despite the widespread use of BCG as a TB vaccine, TB remains the leading cause of death from an infectious agent worldwide (71). Therefore, imitating natural *Mtb* infection through enhancing the efficacy of BCG and its administration at the neonatal period may induce protection against asthma and end this controversy.

In general, the conventional method to increase the efficacy of vaccines is the booster dosing (72), since repeated exposure to both antigen and polarizing cytokine is required for an effective immune response (73). In this regard, the BCG efficacy ranges between 0-80% and wanes over time (29), and it is classified as moderately effective vaccine (74). In addition, repeated intradermal injection of live attenuated vaccine is not acceptable due to the associated adverse effects. Thus, intranasal administration of recombinant immunodominant proteins is a better option (75). Antigen 85A (Ag85A) is a major immunodominant secretory protein in both *Mtb* and BCG (76), and can be incorporated with Mtb32 protein (an immunodominant mycobacterial protein) into an adenoviral vector, as its intranasal administration to asthmatic mice significantly suppresses allergic airway inflammation compared to BCG effect. This suppression occurs *via* increased IFN-γ and IL-10 and decreased IL-4, IL-5, IL-13 and IL-33 (77). In addition, antigen 85B (Ag85B), which is another major secretory mycobacterial protein, was recombined with pMG plasmid and administrated nasally to asthmatic mice inducing a protective effect by increasing IFN-γ/IL-4 ratio (75). Moreover, general *Mtb* secretory proteins, particularly Ag85A, have showed promising results for TB prevention in animals and humans and may replace the primary vaccine in the near future (76, 78, 79). It is expected that developed countries will replace the primary BCG vaccine with the recombinant protein vaccine because they cannot see any necessity for injecting live attenuated BCG concurrent with their low TB prevalence (60). However, there is a growing need for further investigation of intranasal use of Ag85A and Ag85B in humans. Additionally, intranasal administration of recombinant BCG producing genetically detoxified S1 subunit of pertussis toxin can prevent murine allergic asthma by increasing IL-12 and IFN-γ (80). Another method to increase BCG efficacy is the combination of BCG with CpG oligodeoxynucleotide, which is a synthetic TLR9 agonist that stimulates both macrophages (classical type) and DCs with subsequent induction of Th1 response. This combination inhibits allergic inflammation in murine asthma compared to BCG alone and may be a protective candidate effective for allergic asthma (34). In addition, it induces more protection against murine TB compared with BCG alone (81). Likewise, recombinant BCG strains producing IL-12 or IL-18 can further mediate protection against murine allergic asthma by switching from a Th2 response to a Th1 response (82, 83). All these genetic engineering-based modifications of BCG can improve its efficacy and provide enhanced protection against murine allergic asthma when administrated at early age. Thus, their use as boosters or as primary candidate vaccines for the prevention of human atopic asthma is promising for preventing one of the most prevalent chronic disease in the world and must be considered in future research.

## Helminths and Allergic Asthma

Helminths have infected human for thousands of years (84). At present, it is estimated that approximately 30% of the world is infected with at least one species of helminths particularly in poor and less developed communities. There are several types of helminths; the most common worldwide are intestinal nematodes including *Ascaris lumbricoides* (*A. lumbricoides*), *Trichuris trichiura* (*T. trichiura*) and hookworm and schistosomes (9). Basically, helminthic infection activates Th2 cells that secrete IL-4, IL-5 and IL-13, then IL-4 stimulates B cells to produce helminth-specific Ig-E, which opsonize the helminths and promote binding to IL-5 activated eosinophils *via* FcϵR. Eosinophils release their granular contents including the main primary and cationic proteins which destroy the helminths, besides binding to FcϵR on mast cells inducing inflammatory response through the release of vasoactive amines, the production of inflammatory cytokines e.g. tumor necrosis factor (TNF) and lipid mediators that contribute to anti-helminths responses. In addition, IL-4 and IL-13 induce mucus secretion and peristalsis that promotes the expulsion of helminths from the mucosal organs (barrier immunity) (10, 85). On the contrary, helminth infection, also, induces T and B regulatory cells that suppress the immune response through the secretion of IL-10, and transforming growth factor (TGF)-β. This process results in a state of hypo-responsiveness which enables organisms to overcome host resistance, and allows chronic infections (10, 86). However, these regulatory mechanisms may protect the host from an excessive immune response against helminths, repair damage that occurred during migration and feeding of these helminths and enhance resistance to further colonization (9, 87).

In experimental murine allergic asthma (**Table 3**), early and chronic infection of different species of helminths e.g., *Schistosoma mansoni* (*S. mansoni)* (88–91), *Schistosoma japonicum* (*S. japonicum)* (92), *Trichinella spiralis* (*T. spiralis)* (93, 94), *Heligosomoides polygyrus* (*H. polygyrus)* (95, 96), *Litomosoides sigmodontis* (97), *Nippostrongylus brasiliensis* (*N. brasiliensis)* (99) and *Strongyloides stercoralis* (*S. stercoralis)* (98), can prevent disease progression. Also, administration of helminth eggs, particularly *S. mansoni* (90, 100), mediates the same effects. In addition, adoptive transfer of immune cell from infected animals such as *S. mansoni*-induced B regulatory cells (101, 102), *S. japonicum*-induced DCs (103) and *H. polygyus*-induced B cells (104), can protect against murine asthma. With regard to humans, in general, variable helminthic infections among different countries can reduce the risk of developing atopic diseases (105–112). Furthermore, the increased allergic reactivity following long term anti-helminths treatment, supports the hypothesis of the protective role conferred by helminths in atopic diseases (113, 114). Concurrently, there is an inverse correlation between human allergic asthma and helminth infections (**Table 4**) of various species such as *S. mansoni* (115–117), *A. lumbricoides* (24, 117–119), *Necator americanus* (*N. americanus)* (hookworm) (117–120), and *Entrobius vermicularis* (*E. vermicularis)* (pinworm) (121). This reverse association can be attributed to the protective effect against the development of allergic asthma.

**Table 3:** Impact of helminths infection on experimental murine allergic asthma.

Helminth type	Animal type	Animal age	Study times		The proposed effect	Proposed immune mechanisms of suppression compared to asthmatic mice	Study year	References
			Time of infection	Asthma induction times				
*S. mansoni*	BALB/c mice	6-8 weeks	Day 0	- OVA sensitization: days 84 and 98 - Challenge: days 112-114	Preventive	↓ pulmonary inflammation, eosinophilia And AHR ↑ IL-4 and IL-13 ↓ IL-5 - No change in IFN-γ ↑ IL-10 by Bregs	2006	(88)
			Day 35					
*S. mansoni*	C57/Bl6 mice	6 weeks	Day 0	- OVA sensitization: days 91 and 98 - Challenge: day 109	Preventive	↓ pulmonary inflammation, eosinophilia, IL-4, IL-13 and AHR - No change in OVA-specific IgE ↓ IFN-γ - The effect depended on T and B regs	2007	(89)
			Day 28			↓ pulmonary inflammation, eosinophilia, IL-4, IL-13 and AHR - No change in OVA-specific IgE ↓ IFN-γ ↑ IL-10		
			Day 56		No effect			
*S. mansoni*	BALB/c mice	6–8 weeks	Day 0	- OVA sensitization: days 35 and 49 - Challenge: days 56-60	preventive	↓ pulmonary inflammation, eosinophilia, IL-4, IL-5 and OVA-specific IgE - No changes in Tregs nor IL-10	2009	(90)
*S. mansoni* eggs			Day 42		Preventive and/or therapeutic	↓ pulmonary inflammation, eosinophilia, IL-4, IL-5 and OVA-specific IgE ↑ Tregs and IL-10 - The effect didn’t depend on IL-10		
*S. mansoni*	BALB/c and C57BL/6 mice	6-8 weeks	Day 0	- OVA sensitization: days 37, 51 and 58 - Challenge: days 63-65	Preventive	↓ pulmonary inflammation, eosinophilia, IL-5, IL-13, OVA-specific IgE and AHR - The effect depended on Tregs	2013	(91)
			Day 37	- OVA sensitization: days 37, 51 and 58 - Challenge: days 63-65	No effect			
			Day 37	- OVA sensitization: days 37, 51 and 58 - Challenge: days 99-101				
*S. japonicum*	BALB/c mice	5-7 week	Day 0	OVA sensitization: days 24, 38 and 45 Challenge: days 52-54	Preventive	↓ pulmonary inflammation, IL-4, IL-5 and OVA-specific IgE ↑ IL-10	2008	(92)
*T. spiralis*	C57BL/6 mice	5 weeks	Day 0	- OVA sensitization: days 28, 29, 34 and 35 - Challenge: days 41, 42, 47 and 48	preventive	↓ pulmonary inflammation, eosinophilia, IL-5 and AHR - No change in IFN-γ ↑ Tregs, IL-10 and TGF-β	2011	(93)
*T. spiralis*	BALB/c mice	6-10 weeks	Day 0	- OVA sensitization: days 25 and 30 - Challenge: days 40-42	Preventive effects increase as infection progress from the acute to the chronic	↓ pulmonary inflammation, eosinophilia, IL-4, IL-5 and OVA-specific IgE ↑ Total IgE ↑ Tregs	2013	(94)
			Day 20			↓ pulmonary inflammation, eosinophilia, IL-4 and IL-5 ↑ total IgE - No change in OVA-specific IgE ↑ Tregs		
			Day 37			↓ Eosinophilia - No changes in OVA-specific IgE nor total IgE ↑ Tregs		
*H. polygyrus*	BALB/c mice	6 weeks	Day 0	- OVA sensitization: days 14 and 21 - Challenge: days 28 and 30	preventive	↓ pulmonary inflammation, eosinophilia, OVA-specific IgE and AHR ↑ Tregs - The effect depended on IL-10	2006	(95)
*H. polygyrus*	BALB/c mice	6-10 weeks	0	- OVA sensitization: days 0 and 14 - Challenge: days 28 and 29	Preventive and/or therapeutic	↓ pulmonary inflammation, eosinophilia, OVA-specific IgE and AHR ↑ Total IgE ↑ Tregs	2009	(96)
*Litomosoides sigmodontis*	BALB/c mice	6–8 weeks	Day 0	- OVA sensitization: days 11 and 25 - Challenge: days 39 and 40	preventive	↓ eosinophilia, IL-4, IL-5, OVA-specific IgE and AHR ↓ IFN-γ ↓ IL-10 ↑ Tregs and TGF-β	2016	(97)
*S. stercoralis*	BALB/cByJ mice	6–8 weeks	Days 0 and 14	- OVA sensitization: days 21 and 26 - Challenge: day 33	Possible Preventive	↓ Eotaxin and OVA-specific IgE ↑ IL-5 and total IgE ↓ IFN-γ	2000	(98)
*N. brasiliensis*	C57BL/6 and BALB/c mice	5-7 weeks	Day 0	- OVA sensitization: days 32 and 46 - Challenge: day 56	preventive	↓ Eosinophilia and Eotaxin - No changes in IL-5, IL-13 nor OVA-specific IgE - The effect depended on IL-10	2004	(99)
			Day 28		preventive	↓ Eosinophilia and Eotaxin - No changes in IL-5, IL-13 nor OVA-specific IgE ↑ Th2 cells - The effect depended on IL-10		
			Day 49		No effect			

The possible effects of BCG are divided in to 3 categories: (1) preventive, (2) preventive and/or therapeutic and (3) therapeutic; according to 3 time sets of vaccine administration: (1) before allergen sensitization, (2) with allergen sensitization, (3) with/after aerosol allergen challenge respectively. H. polygyrus, Heligosomoides polygyrus; N. brasiliensis, Nippostrongylus brasiliensis; S. japonicum, Schistosoma japonicum; S. mansoni, Schistosoma mansoni; S. stercoralis, Strongyloides stercoralis; T. spiralis, Trichinella spiralis.

**Table 4:** Impact of helminth infections on human allergic asthma.

Country	Study design	Type of helminths	Age of studied population	No. of infected subjects/total subjects		Proposed effect against asthma		Notes	Study year	Reference
Brazil	prospective	*S. mansoni*	6-35 years	41/84		Inverse association		The frequencies of symptoms, use of antiasthma drugs, and pulmonary abnormal findings at physical examination were reduced with infection	2003	(115)
Brazil	case control	*S. mansoni*	6-40 years	33/43		Inverse association		Decreased levels of Th2 cytokines by probable action of IL-10	2004	(116)
Brazil	longitudinal ecological	*S. mansoni*	5-64 years	information from all the 5565 Brazilian municipalities were analyzed		Inverse association		lower asthma hospitalization rates and lower asthma morbidity with infection	2014	(117)
		*A. lumbricoides*								
		hookworm								
Ethiopia	case-control	*A. lumbricoides*	17-67 years	38	153		Inverse association		(118)	(115)
		*N. americanus*		38						
Ethiopia	nested case-control	*A. lumbricoides*	1-4 years	213	7155		Protective		(119)	(116)
		*N americanus* (hookworm)		58						
Mexico	Ecological study	helminthiasis including ascariasis, taeniasis, filariasis, trichuriasis, enterobiasis, and others	All ages	37.5 million (∼35% of total Mexican population)			Inverse association		2017	(24)
Ethiopia	nested case-control	*N americanus* (hookworm)	>16 years	140	604		Inverse association	Protection against wheeze was most pronounced with hookworm and to a lesser extent ascaris	(120)	(117)
		*A. lumbricoides*		228						
Taiwan	retrospective	*E. vermicularis* (pinworm)	6-12 years	429/3107			Inverse association	No significant difference between infected and non-infected group regarding parental asthma	2002	(121)
China	cross-sectional family-based cohort	*A. lumbricoides*	8-18 years	533/2164			Infection was associated with Increased Risk of Asthma and Atopy		2002	(122)
Ecuador	cross-sectional study	*A. lumbricoides*	5-18 years	2006	4433		No association		(123)	(120)
		*T. trichiura*		1768						
		*Ancylostoma duodenale*		91						
Ethiopia	cross-sectional study	*Ascaris*	5-95 years	1145	7649		No association		(124)	(121)
		hookworm		1014						
		*T. trichiura*		172						
		*Hymenolepsis nana*		151						
		*Taenia spp.*		89						
		*S. stercoralis*		55						
		*E. vermicularis*		20						
Brazil	Cross-sectional study	*A. lumbricoides*	12-30 years	47/113		No association			2006	(125)
Bangladesh	Cross-sectional	*A. lumbricoides*	5 years	230/341		Infection was associated with increased risk of asthma		positive family history for asthma among asthmatic subjects increased significantly compared to healthy subjects	2008	(126)
Cuba	Cross-sectional	*T. trichiura*	4-14 years	126	1320		No association	positive family history for atopy among asthmatic subjects increased significantly	(127)	(124)
		Hookworm		121						
		*A. lumbricoides*		83						
		*E. vermicularis*		36						
Brazil	cross-sectional and nested within a cohor	*A. lumbricoides*	4-11 years	190	1182		No association		(128)	(125)
		*T trichiura*		128						
Norway	two-generation Cohort	*T. canis*	10-45 years (Offspring)	21/264		Infection was associated with increased risk of asthma			2018	(129)
			39-63 years (parents)	30/171		No association				
		*A. lumbricoides*	10-45 years (Offspring)	28/264						
			39-63 years (parents)	50/171						
Brazil	population-based cross-sectional	*T. canis*	6–59 months	104/606		Infection was associated with increased risk of asthma			2007	(130)
Denmark	population-based cohort	*E. vermicularis*	0-14 years	132,383 /924,749		No association			2012	(131)

E. vermicularis, Entrobius vermicularis; N. americanus, Necator americanus; T. canis, Toxocara canis.

Although the Th2 immune responses to helminths and allergic asthma seem to be identical, the helminths-induced responses have different characteristics (9, 10), which may constitute the protective mechanisms against atopic asthma. There are two hypotheses for these mechanisms in mice and human (**Figure 1B**). First, the *regulatory network hypothesis* (the most widely accepted), in which chronic helminth infections induce regulatory immune response in the form of T and B regulatory cells that suppress Th2 cell activation and subsequent allergic inflammation *via* the production of anti-inflammatory IL-10 and TGF-β (10, 86, 102, 132–134). The helminths induction of the adaptive regulatory (T and B) cells may be mediated by the initial interaction of the helminths with innate cells as DCs (103) and macrophages (132) to induce regulatory DCs and M2 macrophages, respectively. Then, the latter cells secrete IL-10 which induces T and B regulatory cells. In addition, these regulatory cells, by secreting IL-10 can inhibit DCs, resulting in an alleviation of allergen presentation by DCs and further inhibition of Th2 cells polarization (132, 134). Moreover, *via* IL-10 and TFG-β, they induce more M2 macrophages (2), which reflects regulatory feedback mechanisms between adaptive and innate cells. Since suppressive activities in some studies are continued with regulatory cells after IL-10 depletion, the regulatory function of T and B cells does not always depend on their signature cytokines. Nonetheless, direct interaction with other immune cells such as Th2 or innate cells may play a significant role in the suppressive functions (90, 104). Furthermore, helminth-induced inhibition can occur even with depletion of adaptive regulatory cells, through direct inhibition of DCs (100), and thus the helminth-induced regulation will find its way through either adaptive or innate systems. Interestingly, in allergic asthma, Tregs are dysfunctional with limited inhibitory activities and may exaggerate the pathogenesis of the disease (10, 135). Consequently, helminths-induced regulatory network to mediate the protective effects against atopic asthma is proposed. The second hypothesis is termed the *saturation hypothesis* and it has been proposed earlier than the first hypothesis, in which helminth infections induce polyclonal IgE that bind to and saturate high affinity FcϵR on mast cells and basophils, preventing binding of aeroallergen-specific IgE and block mast cell and basophils degranulation (**Figure 1B**). This is based on the findings that helminth-induced polyclonal IgE is significantly greater than asthma-induced IgE (11, 98, 132, 133, 136). In addition, helminths may induce specific IgG4 that can interact and compete with aeroallergen-specific IgE, which provides another illustration of the *saturation hypothesis* (136–138). In general, these two hypotheses provide an explanation of the protective mechanisms of helminth-induced immune response against atopic asthma, which also represent the fundamental differences between Th2 immune response for helminths and allergic asthma. Therefore, the helminth response is called the modified immune response type 2 (10, 137, 138). In addition, this protection is associated with chronic helminth infections, since acute infection may exacerbate the atopy (100, 132). Interestingly, the immune response to helminth infections has been hypothesized to be the primary objective of the evolution of type 2 immune response arm, not the allergy (139). Therefore, the immune response to helminths represents the normal side of type 2 arm (sensitivity), while the response to allergens, represents the pathogenic side (hypersensitivity). One simple explanation for helminth-induced suppression of allergy is that the type 2 immune response has a limited capacity to respond, that could be depleted by helminth immunity or allergy. In addition, through competition or splitting efforts, the anti-helminthic response will reduce the allergic reactions (137).

In contrast, based on human studies in different countries (**Table 4**), helminth infections, such as *A. lumbricoides*, Hookworm, *T. trichiura*, *E. vermicularis*, *Toxocara canis* (*T. canis*) and *Ancylostoma duodenale*, do not induce any protective effects against allergic asthma (122–131). Furthermore, *A. lumbricoides* (122, 126) and *T. canis* (129, 130) may increase the risk of developing asthma. Moreover, in asthmatics, experimental hookworm infection neither improve bronchial responsiveness nor other asthma measures (140). Conflicting results concerning the protective effect of helminth infections against human atopic asthma can be attributed to several factors. First, the variation of helminth species among studies (133). However, in the previously mentioned human studies, there was a controversy about *A. lumbricoides* (that showed the most conflicting results), hookworm, *T. trichiura* and *E. vermicularis*, whereas there was no conflict regarding the negative association between *S. mansoni* infection and atopic asthma in human. This may be related to helminth chronicle (the second factor of conflict) (133), and the associated granulomatous inflammation and fibrosis (141). In addition, *S. mansoni* is the most studied helminths in mice demonstrating a protective effect against asthma (88–91, 100, 102). Therefore, acute or light helminth infections do not protect against asthma and may exacerbate the allergic inflammation, while chronic infections are more often associated with protection (100) as previously mentioned in the *regulatory network hypothesis*. Thus, the undetermined course of helminth infections, whether acute or chronic, in the previously mentioned human studies is one of the reasons for the controversy concerning the protective effect against atopic asthma. Third, the undetermined host age when acquiring infection in most human epidemiological studies, is another cause of conflict (133), because the infections should occur earlier than the onset of the allergic sensitization, in order to induce preventive effects. Interestingly, all the previously mentioned animal studies supported the protective effects of helminth infections (88–91, 93, 94, 99, 102, 104) (**Table 3**). The courses of infection and the host age upon acquisition of infection, were clearly determined. This indicates that the early and chronic helminth infections is necessary to protect against the development of experimental allergic asthma. Fourth, most human studies did not compare allergic hereditary risk between the infected and non-infected or asthmatic and non-asthmatic subjects, since the significant difference in atopic family history between these groups may interfere with the proposed effects of helminths in human asthma (130). Only two studies (of non-supportive studies) performed this comparison and found a significant association of atopic family history with the asthmatic group (126, 127). Ultimately, these adverse outcomes may be because some helminths possess allergy-inducing and/or anti-allergic molecules, and thus the effect of helminth in asthma will depend on the predominant secretory molecule. For example, *Ascaris suum* (*A. suum*) contains both the allergenic protein of *A. suum*-3 (APAS-3) that induces/exacerbates allergic reactions and the suppressive protein of *A. suum*-1 (PAS-1) that suppresses asthma (142). This highlights the significance of identifying the helminth effector proteins against asthma, which is the next subject of discussion.

### Helminth-Derived Proteins and Allergic Asthma

It is unlikely that infection with live worms or their eggs could be delivered into children as a vaccine to prevent asthma (132). In order to solve this prospective issue, identification of helminth proteins that mediate anti-allergic properties, followed by the production of similar recombinant proteins that mediate the same immune responses observed in live worm infection would be a better option for developing a future vaccine for allergic asthma (143). Many commercial companies and private entities have produced and marketed helminth-derived molecules for the treatment of inflammatory diseases (144). Therefore, several studies demonstrated the role of different helminthic proteins in the prevention and therapy of murine allergic asthma (**Table 5**). One of these effectual proteins is *S. mansoni* schistosomula (Smteg), extracted from the outer layer of the parasite. Smteg administration reduces eosinophilia, Th2 cytokines and allergen specific-IgE, and these effects can be mediated by a high level of IL-10 secreted by alveolar M2 macrophages and DCs. This indicates a potential preventive and/or therapeutic effects of Smteg protein in asthmatic mice (146). In addition, Sm22·6, which is a soluble protein associated with the tegument of *S. mansoni*, induces the same suppressive effects as Smteg through the induction of IL-10 secreted by Tregs. PIII, also derived from *S. mansoni*, mediates the same effects, but without elevation of IL-10. In addition, *S. mansoni*-derived Sm29 induces suppressive effects; however, lesser than the other two molecules (145). Another schistosome is *S. japonicum* containing the SJMHE1 protein, which is an HSP60‐derived peptide. SJMHE1 administration suppresses the development of murine asthma through inhibiting eosinophilia and Th2 cells and inducing Th1 and Treg cells (149). Likewise, soluble *S. japonicum* egg antigen mediates the same effects without inducing Th1 response (147). While P6, P25, and P30 peptides in SjP40 protein, which is the dominant protein of *S. japonicum* eggs, could prevent murine asthma by activating Th1 and alleviating Th2 cells (148). For *A. lumbricoides*, total protein extracts reduce IL-5 and eosinophilia and induce IL-10 to protect mice against experimental asthma (150). Another type of ascaris is *A. suum* that contains three different components. Each component can prevent murine asthma, namely; total protein extracts (151), pseudocoelomic fluid (152) and PAS-1 (142). They reduce eosinophilic inflammation and Th2 activities through the induction of Tregs and Th1 (142) or inhibition of DCs (152). Interestingly, as mentioned previously, *A. suum* has allergenic protein that can induce and exacerbate allergic reaction (142), indicating that identifying effective anti-allergic components in the worm extracts would be a crucial approach for developing novel preventive strategies. Moreover, soluble extracts of adult worms of *T. spiralis* have more potent suppressive impact on allergic asthma than those of soluble extracts of the larvae muscle of the same helminth (11). Another important consideration is the timing of protein injection, as the effective suppressive action of some helminth derived protein depends on early administration prior to developing asthma, which also indicates the importance of elucidating the preventive and therapeutic potentials of each candidate protein. The excretory/secretory products of *Fasciola hepatica* are a clear example of this indication, the administration of which during experimental murine asthma development, induces significant suppressive effects, while their administration after the establishment of the disease has no apparent effect (165). Another significant protein derived from helminth is AIP-2 secreted by *Ancylostoma caninum* (hookworm). AIP-2 has both preventive and therapeutic potentials on murine asthma through inhibition of DCs and induction of Tregs (164). ES-62, another promising molecule secreted by the filarial nematode *Acanthocheilonema viteae* (*A. viteae)*. ES-62 has preventive and/or therapeutic potentials through the direct inhibition by mast cells FcϵRI-induced release of allergy mediators through blocking of key signal transduction molecules (153, 155). Moreover, several proteins derived from different species of helminths demonstrate preventive or therapeutic abilities against murine allergic asthma, such as *Trichuris suis* (162), *H. polygyrus* (156, 157), *N. brasiliensis* (158), *Caenorhabditis elegans* (161), *Clonorchis sinensis* (160), *Angiostrongylus cantonensis* (163) and *Toxascaris leonine* (159) **(** **Table 5** **)**. Thus, worm-derived proteins might be exploited to prevent allergic asthma, since they suppress asthma through the same mechanisms that are induced with living worm infections without entailing their undesired side effects.

**Table 5:** Impact of helminth-derived proteins on allergic asthma.

Helminths type	Protein type	Animal type	Animal age	Route of administration	Study times		Proposed effect against asthma	Proposed immune mechanisms	Study year	References
					Time of protein administration	Asthma induction times				
*S. mansoni*	Sm22.6	BALB/c mice	6-8 weeks	S.C.	Days 0, 10 and 20	- Sensitization: days 2 and 17 - Challenge: days 24-29	preventive	↓ Allergic inflammation, eosinophilia, IL-4, IL-5 and OVA specific-IgE - No change in IFN-γ ↑ Tregs with high IL-10	2010	(145)
	PIII							↓ Allergic inflammation, eosinophilia, IL-4, IL-5 and OVA specific-IgE - No change in IFN-γ ↑ Tregs without increase of IL-10		
	Sm29						Less preventive than other proteins	↓ Allergic inflammation, OVA specific-IgE without significant changes in eosinophilia, IL-4, IL-5 ↓ IFN-γ ↑ Tregs without increase of IL-10		
*S. mansoni*	Smteg	BALB/c mice	6-8 weeks	i.p.	Day 7	- Sensitization: days 0 and 14 - Challenge: days 21-25	preventive and/or therapeutic	↓ Allergic inflammation, eosinophilia, IL-5, IL-13 and OVA specific-IgE ↑ IL-10 by alveolar macrophages and DCs	2016	(146)
*S. japonicum*	Soluble schistosome egg antigen	BALB/c mice	6-8 weeks	i.v.	Days 0, 7, 14 and 21	- Sensitization: days 0, 7 and 14 - Challenge: days 26-28	Preventive and/or therapeutic	↓ Alergic inflammation, eosinophilia, IL-4 and IL-5 - No change in IFN-γ ↑ Tregs	2007	(147)
*S. japonicum*	P6, P25, and P30 peptides in SjP40 protein	BALB/c mice		Injection in footpad and tail base	Days 0 and 14	- Sensitization: days 7 and 21 - Challenge: days 28- 35	Preventive	↓ Allergic inflammation, eosinophilia, IL-4, IL-5, IL-13, IL-17 and OVA specific-IgE ↑ Th1 and IFN-γ	2016	(148)
*S. japonicum*	SJMHE1 peptide	BALB/c mice	6-8 weeks	I.P.	Days 0, 14 and 28	- Sensitization: days 0, 7 and 14 - Challenge: days 21-27	preventive and/or therapeutic	↓ Allergic inflammation, eosinophilia, IL-4 and Th2 cells ↑ Th1 and IFN-γ ↑ Tregs, IL-10, IL-35	2019	(149)
*Angiostrongylus cantonensis*	a crude extract	BALB/c mice	3–8 weeks	i.p.	Day 0, 14 or 42	- Sensitization: days 21 and 35 - Challenge: days 46-48	More preventive than other helminths particularly at early time	↓ Eosinophilia - No change in IFN-γ	2015	(150)
*A. lumbricoides*					Day 14		Preventive	↓ Eosinophilia and IL-5 - No change in IFN-γ ↑ IL-10		
*Angiostrongylus costaricensis*		C57BL/6 mice	6-8 weeks		Day 14		Preventive	↓ eosinophilia and IL-5 - No change in IFN-γ		
*A. suum*	*A. suum* extract	B10.A or C57BL/6 mice	7-8 weeks	s.c.	Day 0	- Sensitization: day 0 - Challenge: days 14-17	Preventive and/or therapeutic	↓ eosinophilia, IL-5, IL-4, OVA specific-IgE and AHR	2002	(151)
*A. suum*	PAS-1	BALB/c mice and Wistar rats	6-8 weeks	i.p. (day 0) s.c. (day 7) i.n. (days 14 and 21)	Days 0, 7, 14 and 21	- Sensitization with APAS-3 protein: days 0 and 7 - Challenge: days 14 and 21	Preventive and/or therapeutic	↓ Eosinophilia, IL-4, IL-5 and APAS-3 specific-IgE ↑ IFN-γ ↑ IL-10	2005	(142)
*A. suum*	pseudocoelomic fluid	BALB/c and C57BL/6 mice	Not mentioned	i.p	Days 0 and 5	- Sensitization: days 0 and 5 - Challenge: days 14 and 15	Preventive and/or therapeutic	↓ Allergic inflammation ↓ DCs activation - The effect didn’t depend on IL-10	2006	(152)
*A. viteae*	ES-62	BALB/c mice	8 weeks	s.c.	Days 2, 12, 25 and 27	- Sensitization: days 0 and 14 - Challenge: days 14, 25, 26 and 27	Preventive and/or therapeutic	↓ Allergic inflammation and AHR ↓ FcϵRI-induced release of allergy mediators from mast cells by selectively blocking key signal transduction events including phospholipase D–coupled, sphingosine kinase–mediated calcium mobilization and nuclear factor-jB activation	2007	(153)
*A. viteae*	Cystatin-17	BALB/c mice		i.p.	Days 1, 7, 14 and 21	Sensitization: days 0 and 14 - Challenge: days 28 and 29	Preventive and/or therapeutic	↓ Allergic inflammation, eosinophilia, IL-4, OVA specific-IgE and AHR ↑ IL-10 by macrophages	2008	(154)
					Days 21, 23 and 25		Therapeutic			
*A. viteae*	11a and 12b (small molecule analogues of ES-62)	BALB/c mice	6-8 weeks	s.c.	Days 0, 14, 27 and 29	- Sensitization: days 2 and 16 - Challenge: days 27-29	preventive	↓ Mast cell degranulation and its cytokine production ↓ Allergic inflammation, eosinophilia and IL-4	2014	(155)
				i.n.	Days 27, 28 and 29		Therapeutic	↓ Neutrophilia, allergic inflammation and IL-13 ↑ IFN-γ		
*H. polygyrus*	*H. polygyrus* excretory-secretory	BALB/c mice	Not mentioned	i.p.	Days 0 and 14	- Sensitization: days 0 and 14 - Challenge: days 28-30	Preventive	↓ Allergic inflammation, eosinophilia, IL-4, IL-5, IL-13 and OVA specific-IgE ↓ IFN-γ and IL-17 ↓ Innate lymphoid cells Type II (ILC2) - No change in Tregs	2012	(156)
				Intratracheal	Days 28-30		Therapeutic	↓ Eosinophilia, IL-5 and ILC2 - No changes in Th1 nor Tregs		
*H. polygyrus*	*H. polygyrus* excretory-secretory	BALB/c, C57BL/6	Not mentioned	i.n.	Day 0	- Sensitization: day 0 - Challenge: days 14-16	Preventive and/or therapeutic	↓ Allergic inflammation, eosinophilia, IL-4, IL-5 and Th2 cells ↓ IL-33 and ILC2 functions - No changes in IFN-γ nor Tregs	2014	(157)
*N. brasiliensis*	excretory– secretory products	C57BL/6 mice	6-8 weeks	I.p.	Days 0 and 14	- Sensitization: days 0 and 14 - Challenge: day 24	Preventive and/or therapeutic	↓ Allergic inflammation, goblet cell hyperplasia, eosinophilia, IL-4, IL-5, OVA specific-IgE and AHR - No changes in IFN-γ nor IL-10	2007	(158)
*Toxascaris leonina*	ES and TP	BALB/cBY mice	6 weeks	i.p.	Days 0 and 7	- Sensitization: days 14 and 21 - Challenge: days 27, 28, 33, and 34	preventive	↓ Allergic inflammation, IL4, AHR (with both ES and TP) and IL-5 (with TP only) - No change in IFN-γ (with both ES and TP) ↑ IL-10 (with TP only)	2008	(159)
					days 27, 28, 33, and 34		Weak therapeutic effect	↓ Allergic inflammation and IL-4 (with TP only) - No changes in IL-5, IFN-γ nor IL-10 (with both ES and TP)		
*Clonorchis sinensis*	*Clonorchis sinensis*-derived total protein	Balb/c mice	5-6 weeks	i.p.	Day 0	- Sensitization: days 1 and 8 - Challenge: days 15-18	Preventive	↓ AHR, allergic inflammation, eosinophilia, IL-5, IL-13 and OVA specific-IgE ↓ DCs activation ↑ Tregs and IL-10	2011	(160)
					Day 14		therapeutic			
*Caenorhabditis elegans*	Crude Extracts	BALB/c mice	7 weeks	i.p.	Days 0 and 7	- Sensitization: days 0 and 7 - Challenge: days 14, 15, 21 and 22	Preventive and/or therapeutic	↓ Allergic inflammation, eosinophilia, IL-4, IL-5, IL-13, OVA specific-IgE and AHR ↑ IFN-γ and IL-12 No change in IL-10	2012	(161)
				i.n.	1 dose (50 or 10 μg) at day 28 or 4 doses (25 μg) at days 28, 35, 42, and 49		therapeutic			
*Trichuris suis*	excretory/secretory products	BALB/c and C57Bl/6J mice	8 weeks	i.p.	days 0, 7, 14 and 21	- Sensitization: days 0, 14 and 21 - Challenge: days 28 and 29	Preventive and/or therapeutic	↓ Allergic inflammation, eosinophilia, IL-4, IL-5, IL-13, OVA specific-IgE and AHR - The effect was partially mediated through IL‐10	2014	(162)
*Angiostrongylus cantonensis*	Recombinant AcCystatin protein	Wistar rats	8 weeks	i.p.	Day 0	- Sensitization: days 1 and 8 - Challenge: day 15	Preventive with more suppressive action than the therapeutic effect through more production of IL-10	↓ Allergic inflammation, eosinophilia, IL-4, IL-5, IL-17 and OVA specific-IgE - No change in IFN-γ ↑ IL-10	2015	(163)
					Day 14		therapeutic	↓ Allergic inflammation, IL-4, IL-5 and and OVA specific-IgE ↑ IL-10		
*Ancylostoma caninum* (hookworm)	anti-inflammatory protein-2 (AIP-2)	BALB/c.ARC and C57Bl/6 mice	3-12 weeks	i.n.	Days 12-15	- Sensitization: days 0 and 7 - Challenge: days 18-22	preventive	↓ Allergic inflammation, mucus production, collagen deposition, IL-5, IL-13, OVA specific-IgE and AHR ↓ DCs activation and proliferation ↑ Tregs	2016	(164)
					Days 20-24		Therapeutic			
*Fasciola* *hepatica*	excretory/secretory products	BALB/c Mice	6-12 weeks	i.p.	Days 0 and 14	- Sensitization: days 0 and 14 - Challenge: days 24-26	Preventive	↓ Allergic inflammation, eosinophilia, IL-4, IL-5 and IL-13	2017	(165)
					Days 24-27		No effects			
*T. spiralis*	soluble extracts of adult worms	Balb/c mice	6-8 weeks	i.p.	(Group I) Days 0, 7 and 14	- Sensitization: days 21, 35 and 42 - Challenge: days 49-51	preventive	↓ Allergic inflammation, eosinophilia, IL-4, OVA specific-IgE and AHR - No change in IFN-γ ↑ TGF-β - The preventive effect was greater than the therapeutic through more reduction of OVA specific-IgE	2019	(11)
					(Group II) Days 21, 35 and 42		preventive and/or therapeutic			
	soluble extracts of muscle larvae				As group I		Less suppressive effects than those of the extracts of adult worms	↓ Allergic inflammation and IL-4 ↑ TGF-β		
					As group II					

The possible effects of helminth-derived proteins are divided in to 3 categories: (1) preventive, (2) preventive and/or therapeutic and (3) therapeutic; according to 3 time sets of vaccine administration: (1) before allergen sensitization, (2) with allergen sensitization, (3) with/after aerosol allergen challenge respectively. A. lumbricoides, Ascaris lumbricoides; A. suum, Ascaris suum; A. viteae, Acanthocheilonema viteae; AIP-2, anti-inflammatory protein-2; ES, Excretory/secretory protein; H. polygyrus, Heligosomoides polygyrus; N. brasiliensis, Nippostrongylus brasiliensis; S. japonicum, Schistosoma japonicum; S. mansoni, Schistosoma mansoni; Smteg, Schistosomula tegument; T. spiralis, Trichinella spiralis; TP, Total protein.

There are many studies investigating the protective effects of helminth-derived proteins in murine allergic asthma. Nevertheless, there are only few studies that detected these protein actions in human allergic asthma. *S. mansoni* antigens, Sm29 and Sm29TSP-2 reduce Th2 cells while inducing Tregs with subsequent high IL-10 production in the cell cultures from asthmatic patients (166). In same vein, other *S. mansoni* recombinant antigens as Sm22.6, Sm14, P24, and PIII antigen increase IL-10 secretion in cell cultures from subjects with asthma (167). Additionally, AIP-2, from the hookworm, suppresses human DCs activation and proliferation from asthmatic subjects *in-vitro* (164). In addition, ES-62 from *A. viteae* when co-cultured with sensitized human mast cells from healthy subjects, mediates the same effects previously mentioned with ES-62 with murine mast cells (153). However, these studies only demonstrate *in-vitro* therapeutic potentials for human asthma. Therefore, further research is needed to investigate both *in-vitro* and *in-vivo* preventive potentials of helminth-derived proteins against the development of human allergic asthma, to design novel strategies for potential allergic asthma vaccines.

## Summary and Conclusions

In this study, we discussed the immunomodulatory effects of different natural infections on the development of asthma, and the benefits of imitating these phenomena *via* the use of effective proteins-based vaccines for future disease control. Natural infection with *Mtb* prevents the development of allergic asthma. Therefore, BCG vaccine is suggested at an early age to mediate the same prevention particularly with increasing its efficiency through genetic engineering-based modifications, which are beneficial for tuberculosis prevention as well. Similarly, natural helminthic infections may prevent allergic asthma development. Therefore, helminth-derived protein at early age is an effective candidate for designing allergic asthma vaccines and requires further investigation. We revealed the beneficial features of the *hygiene hypothesis* for preventing allergic asthma *via* simulating the natural infections that either induce Th1 or Th2 cells primarily as nature will find its way regardless of the immune pathway, and this can also be applied to other allergic diseases. In addition, since the *hygiene hypothesis* also includes autoimmune diseases as mentioned earlier, imitation of the nature could be the missing key for such diseases. We, therefore, recommend mimicking nature to be a general strategy for preventing allergic asthma and other diseases that increased dramatically over the past decades.

## Author Contributions

MA conceptualized the idea. MA, XJ, JW and FA performed the literature study and wrote the original draft. MA, SA and HX discussed, reviewed and edited the manuscript. All authors contributed to the article and approved the submitted version.

## Funding

This work was supported by National Natural Science Foundation of China (Grant No. 81771756) and the Doctorial Innovation Projects of Jiangsu Province (Grant no. KYCX17_1816).

## Conflict of Interest

The authors declare that the research was conducted in the absence of any commercial or financial relationships that could be construed as a potential conflict of interest.

## Publisher’s Note

All claims expressed in this article are solely those of the authors and do not necessarily represent those of their affiliated organizations, or those of the publisher, the editors and the reviewers. Any product that may be evaluated in this article, or claim that may be made by its manufacturer, is not guaranteed or endorsed by the publisher.

## Abbreviations

*A. lumbricoides*, *Ascaris lumbricoides; A. suum*, *Ascaris suum; A. viteae*, *Acanthocheilonema viteae*; Ag85A, Antigen 85A; Ag85B, Antigen 85B; AHR, Airway hyperreactivity; AIP-2, anti-inflammatory protein-2; APC, Antigen presenting cell; BCG, Bacille Calmette-Guérin; DCs, Dendritic cells; *E. vermicularis*, *Entrobius vermicularis*; ES, Excretory/secretory protein; *H. polygyrus*, *Heligosomoides polygyrus*; i.d., Intradermal; i.n., Intranasal; IL, Interleukin; ISAAC, International Study of Asthma and Allergies in Childhood; *M. bovis*, *Mycobacterium bovis*; Mtb, Mycobacterium tuberculosis bacteria; *N. americanus*, *Necator americanus; N. brasiliensis*, *Nippostrongylus brasiliensis*; OVA, Ovalbumin; RD, Region of difference; *S. japonicum*, *Schistosoma japonicum; S. mansoni*, *Schistosoma mansoni; S. stercoralis*, *Strongyloides stercoralis*; Smteg, schistosomula tegument; *T. canis*, *Toxocara canis; T. spiralis*, *Trichinella spiralis; T. trichiura*, *Trichuris trichiura*; TB, Tuberculosis; TGF, Transforming growth factor; Th, T helper; TLR, Toll-like receptor; TNF, Tumor necrosis factor; TP, Total protein; Tregs, T regulatory cells.
